# Early Stage Alpha-Synuclein Amyloid Fibrils are Reservoirs of Membrane-Binding Species

**DOI:** 10.1038/s41598-018-38271-2

**Published:** 2019-02-11

**Authors:** Thomas Skamris, Carlotta Marasini, Kenneth L. Madsen, Vito Foderà, Bente Vestergaard

**Affiliations:** 1 Department of Drug Design and Pharmacology, Faculty of Health and Medical Sciences, University of Copenhagen, Universitetsparken 2, 2100 Copenhagen, Denmark; 2 Department of Pharmacy, Faculty of Health and Medical Sciences, University of Copenhagen, Universitetsparken 2, 2100 Copenhagen, Denmark; 3 Department of Neuroscience, Faculty of Health and Medical Sciences, University of Copenhagen, The Panum Institute, Maersk Tower 7.5, 2200 Copenhagen, Denmark

## Abstract

The presence of αSN fibrils indisputably associates with the development of synucleinopathies. However, while certain fibril morphologies have been linked to downstream pathological phenotypes, others appear less harmful, leading to the concept of fibril strains, originally described in relation to prion disease. Indeed, the presence of fibrils does not associate directly with neurotoxicity. Rather, it has been suggested that the toxic compounds are soluble amyloidogenic oligomers, potentially co-existing with fibrils. Here, combining synchrotron radiation circular dichroism, transmission electron microscopy and binding assays on native plasma membrane sheets, we reveal distinct biological and biophysical differences between initial and matured fibrils, transformed within the timespan of few days. Immature fibrils are reservoirs of membrane-binding species, which in response to even gentle experimental changes release into solution in a reversible manner. In contrast, mature fibrils, albeit macroscopically indistinguishable from their less mature counterparts, are structurally robust, shielding the solution from the membrane active soluble species. We thus show that particular biological activity resides transiently with the fibrillating sample, distinct for one, but not the other, spontaneously formed fibril polymorph. These results shed new light on the principles of fibril polymorphism with consequent impact on future design of assays and therapeutic development.

## Introduction

Alpha-synuclein (αSN) is a small (14 kDa) intrinsically disordered protein highly expressed in the presynaptic neuron^1^. Although its physiological function is still debated, the current consensus indicates its involvement in regulating neuronal transmission by interacting with the membrane of synaptic vesicles^2^. The link between αSN and neurodegenerative diseases is, however, well established. Specifically, the protein is a major component of the different intracellular aggregates found in the synucleinopathies^3^: αSN-rich Lewy Bodies are found in the brains of diseased patients suffering from Parkinson’s disease (PD) and dementia with Lewy bodies, and αSN is a major component in the glial inclusion bodies found in patients with multiple system atrophy^3–5^. Much effort is therefore put into obtaining a thorough and detailed description of how αSN misfolds, aggregates and matures to form the amyloid fibrils constituting the disease specific aggregate types. Understanding the full physico-chemical nature of fibrils, and the processes by which they form, is paramount in order to develop therapeutic strategies to treat patients.

Historically, the formation of amyloid fibril is interpreted through a 3-stage kinetic model represented by a sigmoidal growth curve^6^. An initial lag-phase is followed by a rapid elongation phase, ending with a plateau, where the fibrils coexist in equilibrium with soluble protein species^7,8^. Thioflavin T (ThT) fluorescence development is the most commonly used assay to detect the formation of cross β-sheet structures, the dominant structure of the fibril core, allowing e.g. for a rough fibril quantification at the plateau, which hence is considered as a semi-stable state^9–11^. In recent years however, numerous studies reporting continuous rearrangements of amyloid fibrils in the plateau phase are beginning to question whether the classical view of fibril equilibrium holds true^12–16^. A contribution to this debate is the phenomenon of fibril polymorphism, where a protein ensemble can give rise to several fibril classes with distinctive morphologies. Different polymorphs with distinct biological characteristics have also been denoted as different ‘strains’ in the literature since individual polymorph types can be both infectious and inheritable^17,18^. Hereafter, we will use the term ‘polymorphs’ to refer to different levels of aggregate maturation and different fibril species. The different polymorphs can co-exist during elongation and the plateau phases, but there are also examples of conversion between polymorphs^19^. Such transformations can involve either inter- or intra-conversions, i.e. either involving protein dissociation from one strain before aggregating into another, or conversely depending only on intra-fibril structural changes. Until recently, polymorphs were distinguished mainly by their overall appearance and shape^20^ and typical examples of different fibril polymorphs originate from comparison of various mutant fibril forms^21,22^. However, in recent years, several studies have also confirmed that different polymorphs originating from the same parent molecule can exhibit structural differences in the secondary, tertiary and quaternary level both *in vitro*^23–26^ and *in vivo*^17,27^. In addition to this, polymorphs with different thermodynamic properties have been reported^28–30^. Several of these studies compare fibrils that have been formed under different experimental conditions. However, fibrils of wt αSN have been shown to undergo conversions after reaching the steady state, detectable by linear dichroism and small angle scattering^21^. A different study demonstrates that wt αSN, having reached the plateau phase of aggregation in Tris buffer, undergoes a slow maturation (>3 months) when stored at 4 °C and does not disintegrate at this temperature^31^. In contrast, αSN fibrils formed in sodium phosphate buffer (PBS) rapidly disintegrated (<4 hours) when incubated at 10 °C^32^. These apparently conflicting results could find an explanation in the intrinsic polymorphism of fibrils. Indeed, fibrils grown under different physico-chemical conditions (stirring vs. sonication, pH, salt concentration and time of the heat treatment) possess different thermal stabilities^32^.

The discovery that a protein ensemble can convert into fibrils with different morphologies, structures and thermodynamic properties directly translates into potentially different physiological pathways with different impacts on the associated diseases, which is a central aspect of the prion disease and prion-like diseases. Different procedures for forming αSN fibrils *in vitro* indeed generate polymorphs causing separate neurotoxic phenotypes when injected into the brains of rats^17^. Whether these different toxic profiles relate directly to the specific features of the different fibril polymorphs, or rather to the presence of different fibril-associated species is currently unknown. However, oligomers and/or pre-fibrillar species are in general acknowledged as more cytotoxic than mature fibrils^33–35^. Indeed, different fibril polymorphs can potentially either act as efficient sequesters of toxic species, thereby protecting cell integrity^36^ or, if unstable, they can release toxic species into the surroundings^37^. These pieces of evidence point towards potential multiple etiologies for the disease progression, and forces one to carefully consider the whole wide spectrum of fibril polymorphs, their physical stability, and their structural relations, when developing therapeutic strategies. In a worst-case scenario, drugs can indeed be specific towards stable polymorphs, while acting as a destabilizer towards less stable species and hence lead to the unwanted cascade release of toxic species in the cellular environment.

Here we show that wt αSN amyloid fibrils formed in PBS buffer at 37 °C undergo a highly dynamic maturation process within 3 days. The maturation process converts thermally unstable fibrils into robust fibrils with a pronounced right-handed twisted β-sheet. We do not detect morphological differences between the two types of aggregates, except for a slight enhanced degree of fibril clustering, typically observed upon maturation of fibrils^38^. When exposed to a temperature-ramp, the early-formed unstable fibrils significantly disintegrate and release structurally disordered species that bind to cellular membranes. This effect is barely detectable for the mature fibrils. Our work shows that early polymorphs can act as reservoirs of membrane-binding species, releasing them upon destabilization. On the contrary, matured and more stable fibrils protect cell integrity by efficiently embedding these soluble species within the fibril core. Our results suggest that the matured fibrils are formed via structural intra-conversions of the early-formed fibrils. Our findings bring about the notion that effective treatments for synucleinopathies are dramatically dependent not only on the early diagnosis of the diseases, but also on a detailed monitoring and distinction of the whole range of co-existing and highly dynamic species.

## Results and Discussion

### αSN undergoes rapid structural maturation during the plateau of ThT-monitored fibril formation

**Figure 1:**
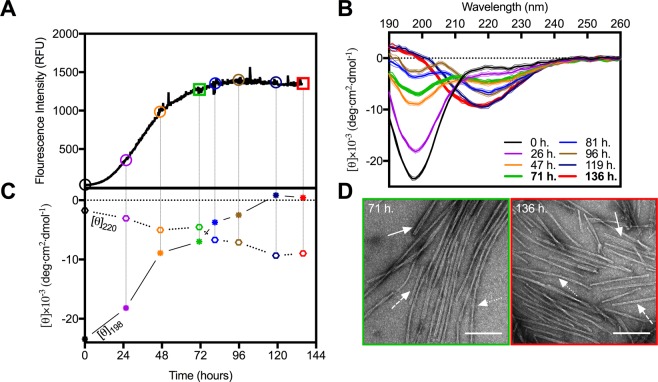
Structural maturation of αSN is not accompanied by noticeable changes in fibril morphology. (**A**) The fluorescent intensity of ThT (λ_ex/em_ = 450/480 nm) during αSN *in vitro* fibrillation. Colored objects indicate the time points at which samples were extracted for SRCD measurements to provide (**B**) corresponding far-UV CD spectra. (**C**) Time-dependent conformational changes of αSN during fibrillation monitored at λ_198nm_ (asterisks) and λ_220nm_ (hexagons). (**D**) Representative TEM images of αSN amyloid fibrils from the early (green) and late (red) phases of the plateau. Solid, dashed and dotted arrows indicate examples of striated ribbon, rod-like and twisted morphologies, respectively. Scale bars represent 200 nm.

In order to follow the conversion of monomeric αSN into β-sheet-rich amyloid fibrils, during *in vitro* fibrillation we used *in situ* ThT fluorescence (Fig. 1A). The signal presented a sigmoidal profile with a lag phase of 17 hours, followed by the elongation phase and a plateau reached within 72 hours. Moreover, at different time intervals (colored circles in Fig. 1A) aliquots of the sample were extracted and measured using synchrotron radiation circular dichroism (SRCD) to provide orthogonal structural information. The collected CD spectra (Fig. 1B) as expected showed a gradual disappearance of the characteristic minimum at 198 nm, corresponding to the random coil of an intrinsically disordered protein (IDP). This was accompanied by a slow appearance of a maximum at 195 nm. The final matured samples reveal the expected minimum around 220 nm indicating the formation of right-handed twisted β-sheet structures^39^. The gradual transition from αSN monomers (black curve, Fig. 1B) to β-sheet-rich structures (red curve, Fig. 1B) is clearly visible when the CD signals at 198 and 220 nm are plotted as a function of time (Fig. 1C).

Notably, the temporal evolution of the ThT intensity did not correlate with the relative amount of β-sheet, reported at 220 nm. Indeed, while the ThT fluorescent signal reached a plateau after approximately 71 hours, the formation of β-sheets further developed until the end of the experiment (approx. 136 hours). Clearly, under these experimental conditions ThT does not faithfully report the further structural changes occurring between 71 and 136 hours or the corresponding change in the equilibrium between species within the sample. Additionally, the CD spectra from the early (71 hours) and the late phase (136 hours) of the plateau (Fig. 1B, green and red curves, respectively) were markedly different. At the early phase there was still a large contribution from unfolded structures (the ellipticity at 198 nm was −7.0 × 10^−3^ deg cm^2^ dmol^−1^) with only a minor contribution from the minimum around 220 nm. The late phase sample varied in both amplitude (more than double) and position (shifted towards 218 nm), while simultaneously demonstrating a maximum at 195 nm, suggesting a significant shift towards β-sheet structures^40^.

To verify the presence of fibrils and to assess whether the structural changes obtained from the SRCD measurements were paralleled with changes in the fibril morphology, we analyzed the αSN samples after 71 and 136 hours of incubation by TEM (Fig. 1D). In both cases, the samples contained large amounts of bundled and isolated fibrils (see Supporting Information Fig. S1) with varying lengths (0.1–5 μm) and morphologies (i.e. striated ribbons, twisted and rod-like). Only minute amounts of amorphous-like aggregates could be identified for both sample types. Within the ensemble of analyzed fibrils, there were no observable differences or clear dominance of one or the other morphology when comparing the two samples. Visible, however, was the increased tendency of fibril clustering at 136 hours. Such clustering has been observed previously for various types of fibrils^38,41–43^, and is normally interpreted as a longitudinal assembly (annealing) of unchanged fibrils, and hence cannot explain the observed changes in the SRCD spectra. We then concluded that the striking observation of structural changes between the early and late phase stages of αSN fibrillation is not detected by the ThT signal, and also not related to any observable macroscopic change in the morphology within the resolution of the electron microscope. However, the high sensitivity of SRCD clearly reveals significant changes of the secondary structural content of early and late fibrils.

### Fibrils from the early plateau are more thermodynamically unstable than in the late plateau

**Figure 2:**
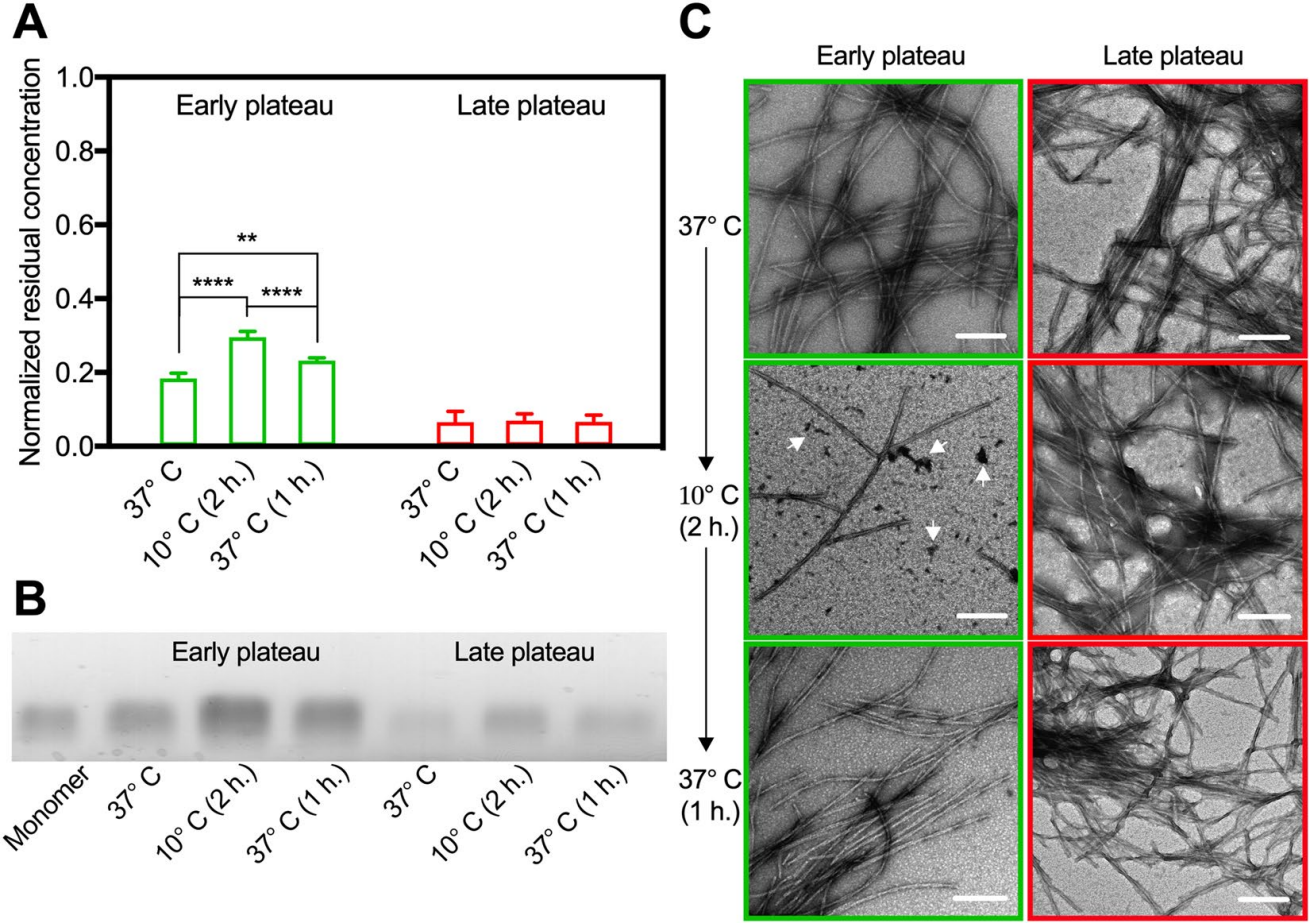
Early plateau fibrils readily disintegrate while late plateau fibrils remain intact. (**A**) The residual concentration (means ± s.d., n = 5) of the supernatant, from samples extracted in the early (green) or late (red) phase of the plateau, normalized to the concentration prior to fibrillation. Samples were centrifuged (1 hour at 14,500 rpm) and measured immediately after extraction and after 2 hours incubation at 10 °C with or without subsequent incubation at 37 °C for 1 hour (two-way ANOVA, Tukey Test; **P < 0.0021, ****P < 0.0001) (**B**) Migration bands after native gel electrophoresis of monomeric αSN prior to fibrillation (left) and of the supernatant samples corresponding to panel (**A**). The gel has been cropped, and the full gel is shown in Supplementary Fig. S3. (**C**) Representative TEM images of the incubated samples prior to centrifugation. Arrowheads highlight amorphous aggregates. Scale bars are 200 nm.

It is well established, that the extensively used ThT-assay for detection of the formation of amyloid cannot be used quantitatively without caution^44^. Fibril polymorphs may bind different amounts of ThT and/or cause different fluorescence intensity, and intermediate, non-fibril aggregates may also contribute to the recorded fluorescence intensity. In consequence, the here observed evolution of the secondary structure signal within the plateau (71 hours < t < 136 hours) can have three possible explanations: (1) a continuous depletion of monomers and production of intermediate soluble species and fibrils, (2) a maturation of already existing fibrils (which is not manifested into observable differences by TEM or ThT signal) or (3) a combination of the two. We hence centrifuged the samples for 1 hour at 14,500 rpm and measured the residual concentration of the collected supernatant (Fig. 2A).

Indeed, we observed a large difference in the residual concentration between the early and late phase (18 ± 1 vs. 7 ± 3%). This is in good agreement with the observation from the SRCD data, that there is still a substantial amount of disordered protein present at the early stage, which is nearly depleted at the late stage (Fig. 1B,C). It is therefore evident that the structural difference between the early and the late phase of the plateau can in part be explained by a continuous conversion of soluble protein, with a predominant random coil structure, into high molecular weight (HMW) and β-sheet-rich fibrils, but it remains possible that an additional maturation of the early formed fibril material can take place simultaneously.

It is sensible to believe that a difference in secondary structure will be reflected by differences in thermodynamic properties. Hence, in order to further investigate the differences between the early and late plateau fibril samples, we looked at their thermal stabilities. Ikenoue *et al*. have demonstrated how polymorph preparations of αSN fibrils formed by stirring are sensitive to heat (60–110 °C) and cold-denaturation (0–20 °C) and quickly reassemble when temperature is returned to 30–50 °C^32^. Inspired by these findings, we evaluated the changes in residual concentration of soluble species in fibrillated αSN from the early (71 hours) and late phase (136 hours) after incubation at 10 °C for 2 hours and again after reheating to 37 °C for 1 hour. If the secondary structure, and hence the physico-chemical stability of the samples is different, we should expect a different thermodynamic behavior when exposed to this three-step thermal gradient. For the early phase samples, the treatment at lower temperature caused the concentration of soluble protein to nearly double (30 ± 2%), while returning the sample to 37 °C for an hour led to a consecutive decrease (23 ± 1%). Remarkably, the residual concentration of the late phase samples was unchanged between the three conditions (7 ± 3, 7 ± 2 and 7 ± 2%). Hence, the equilibrium between soluble species and fibrils in the early phases of the plateau could be altered by changing the temperature from 37 to 10 °C in the timescale of a few hours. This was no longer the case further into the plateau (136 hours), where the CD signal displayed dominant β-sheet features. Our data clearly show that prolonged incubation of fibrils at 37 °C after the nominal ThT plateau is reached (71 hours) not only leads to a further conversion of soluble protein into fibrils, but also to a further structural maturation of fibrils yielding increased stability.

We further characterized the supernatants from the thermal treatments of early and late plateau samples, respectively. Native gel electrophoresis confirms that significantly less protein is co-existing with late plateau samples, relative to the substantially larger fraction of soluble protein present in samples from early plateau samples (Fig. 2B). Of further interest, it is evident from the native gel analysis, that the soluble species, released from early plateau fibril samples, result in a significant smearing of the gel band, hence indicating the presence of a distribution of soluble species of different sizes. The position of the smear is overlapping with the band of the monomeric sample (included in Fig. 2B), but clearly also includes lesser migrating species, suggesting that the soluble fraction, co-existing with early plateau fibrils, could include oligomeric species. Although the presence of soluble oligomers is not strictly conclusive, the observation is of evident interest, in the light of the alleged cytotoxicity of soluble pre-fibrillar oligomers^33–35^. Further substantiating these findings, analysis by dynamic light scattering reveals that the distribution of calculated hydrodynamic diameters from the soluble fraction of early plateau fibrils is shifted towards larger values, compared to the monomeric αSN sample (Supporting Information Fig. S4). The concentration of the soluble proteins from the late plateau samples was too low to allow for a similar analysis, and the raw data indicate the presence of larger particles in the size-range of fibril species (Fig. S4) although these species are not of a concentration high enough to be visible on the native gel (Fig. S3).

We then proceeded to analyze the same samples by TEM (Fig. 2C). While the only discernible distinction between the samples prior to the cooling procedure was an increase in fibril clustering, there were notable differences between the two stages after the incubation at 10 °C. While the late plateau sample after incubation at 10 °C (Fig. 2C, right middle) was indistinguishable from the sample taken directly from 37 °C (Fig. 2C, right top), the fibril amount from the early plateau phase reduced appreciably after incubation at 10 °C (Fig. 2C, left top and middle). Moreover, intensely stained amorphous-like aggregates were also clearly visible (arrows in Fig. 2B, left middle and Supporting Information Fig. S2). Interestingly, the general appearance of those aggregates was similar to the recently reported amorphous-like oligomers formed upon disintegration of αSN fibrils in the presence of the plant extract *Geum urbanum*^45^. It is therefore further likely that the observed increase in the amount of soluble protein (Fig. 2A) was due to the disintegration of fibrils. Furthermore, amorphous-like aggregates were only observed for the early plateau sample incubated at 10 °C and were no longer present when the sample had been reheated to 37 °C. This suggests that the release of soluble species, appearing as amorphous-like aggregates on the TEM grids, is reversible, quite in agreement with the results from Ikenoue *et al*. Even if reduced, when compared to the sample at 37 °C, the amount of fibrils (bundles and isolated) was still substantial at 10 °C (Fig. S2). Moreover, the heterogeneity of fibril morphologies and lengths appeared unaltered. Based on the TEM analysis, it was not possible to identify a particular fibril morphology that was absent after cooling. Together, the results showed that there were marked structural differences between the early and late plateau of fibrillation, and that these differences were accompanied by distinct thermodynamic behaviors. Indeed, in the light of the hypothesis that the alleged toxicity of fibrillogenic material resides with soluble amyloidogenic species rather than the fibrils themselves, it is highly interesting, that immature fibrils release such reversibly soluble species, while the mature fibrils do not.

### The release of structurally disordered species upon temperature changes is fully reversible for early phase fibrils

**Figure 3:**
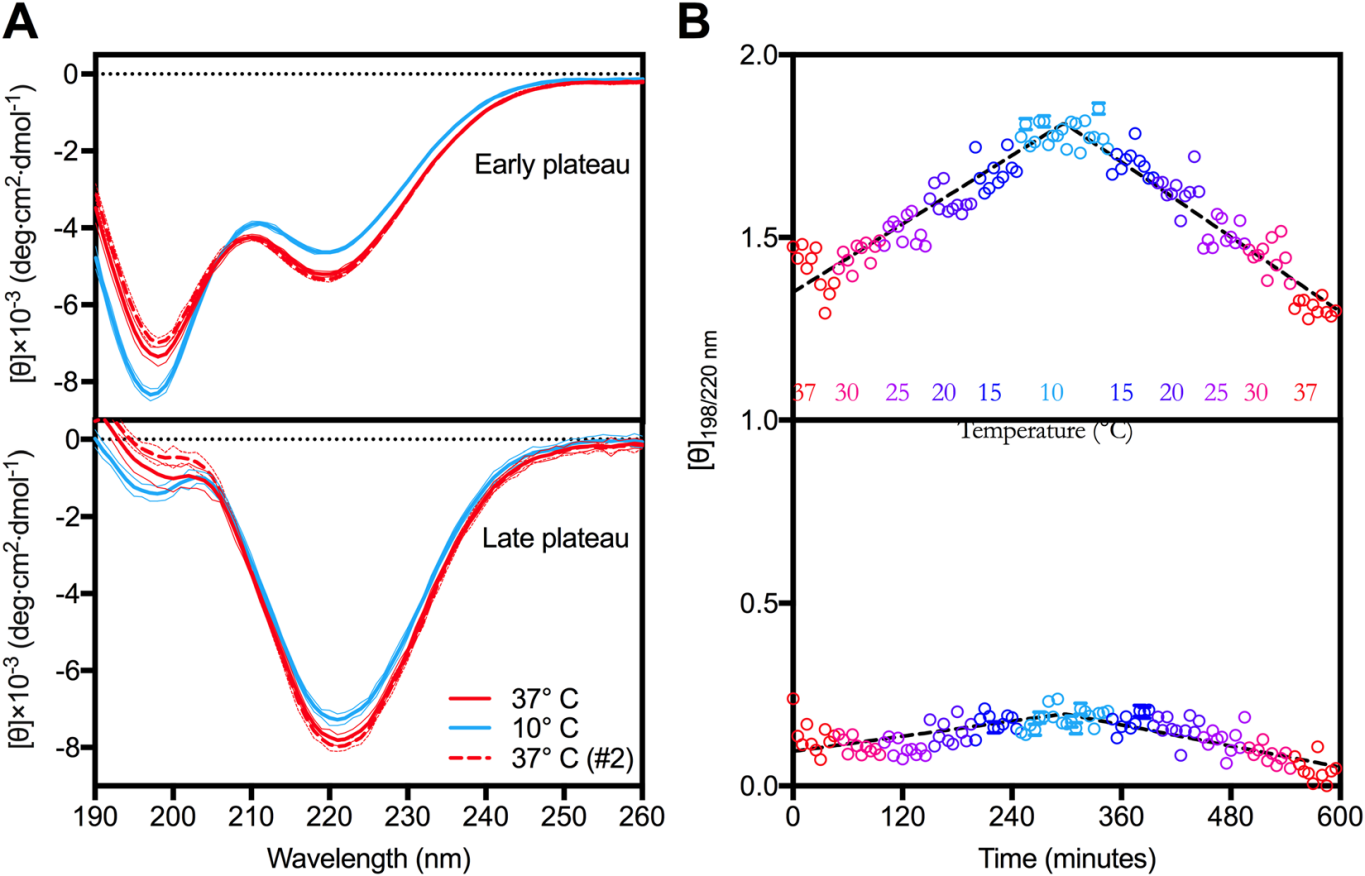
Early plateau fibrils are in thermodynamic equilibrium with structurally disordered species. (**A**) Averaged CD spectra of early (top) and late (bottom) phases of the plateau at 37 °C (red, solid), 10 °C (blue, solid) and again at 37 °C (red, dashed). (**B**) The [θ]_198/220 nm_ ratio ± standard deviation of the ellipticities from individual frames during cooling (37-30-25-20-15-10 °C) and heating (10-15-20-25-30-37 °C) cycles of early plateau (top) and late plateau (bottom) samples have been calculated and plotted as function of time with colors ranging from red to light blue.

We reemployed SRCD to verify whether the above-mentioned fibril disintegration was associated with a restoration of random-coil structures. CD spectra of early and late plateau samples were consecutively collected during a gradual temperature cycle from 37 °C to 10 °C and back to 37 °C (Fig. 3A, see materials for details).

The early plateau sample at 37 °C displayed the same characteristics as seen from the previous analysis (Fig. 1B). Although displaying the typical β-sheet signal at 220 nm, there was still a pronounced minimum at 198 nm, corresponding to a significant proportion of random coil. When the temperature was lowered, the minimum at 198 nm intensified and it was indeed accompanied by a corresponding reduction in the sample β-sheet content as detected by the decreasing signal at 220 nm. Hence, the occurrence of soluble protein at low temperatures is, at least in part, caused by fibril disintegration and loss of secondary β-sheet structures. This also supports the qualitative observation by TEM of reduced fibril material in the 10 °C sample (Fig. 2B). When the sample was brought back to 37 °C, the random coil contribution diminished and β-sheet structures reformed, providing an almost perfect spectral overlap with the initial 37 °C state (solid and dashed red in Fig. 3A).

The late phase plateau sample showed a contrasting behavior. The initial sample revealed the expected deeper minimum at 220 nm and an only minor shoulder around 198 nm (Fig. 3A, bottom). When cooled to 10 °C, the late stage fibrils also displayed signs of destabilization, although these are to a significantly lesser extent than their counterparts at the early plateau phase. To directly inspect and compare the changes with temperature for the two plateau phases, we calculated the ellipticity ratio at 198/220 nm for each individual measurement and plotted them as function of time (Fig. 3B). Here, an increase in [θ]_198/200 nm_ ratio corresponds to the release of disordered species. Using this representation, it is clear how pronounced the disintegration of fibrils into disordered species is during the cooling of the early phase fibrils. The amplitude of change directly relates to relative amounts of random coiled species formed at the cost of β-sheet structures. While the ratio increases dramatically during cooling for a sample from the early plateau phase, there are only minor changes for the sample from the late plateau phase (Fig. 3B). As it is directly observable, reheating the sample leads to a corresponding decrease in ratio, which ultimately confirms that the release of soluble and disordered molecules are in a reversible equilibrium with the thermodynamically unstable early phase fibril forms (Fig. 3B, top). Over time however, fibrils will mature and rearrange into structures that are more stable and do not disintegrate to any significant extent under the stressing conditions tested here (Fig. 3B, bottom).

### The released polymorph specific soluble species exhibit membrane specific binding

It is clear from our results that immature fibrils reversibly release soluble species, while mature fibrils do not. According to the strain hypothesis, polymorphic differences could imply corresponding differences in their biological activity. In the present case, it is thus of obvious interest to investigate, whether the specific release of amyloidogenic soluble species from immature fibrils is associated with such potential differences in biological activity. We thus continued with investigating whether the soluble species that form upon cooling of the fibril sample in the early phase interact with the cellular membrane, and hence act as a biological culprit. We employed *in vitro* binding studies of the sample preparations to supported cell-membrane sheets. The experimental setup, where gentle sonication of cultured cells leads to exposure of the basal domain of the cell membrane, has been used previously to study a variety of cellular properties, such as anchored vesicular structures and endocytic buds^46,47^. Here, we demonstrate how it can be utilized to investigate protein binding to native-like membranes using primary immuno-staining. In order to compensate for the long incubation times needed for the immuno-binding experiments (see Methods for details), we extended the cooling (6 hours at 4 °C) and heating (6 hours at 37 °C) time for the early and late plateau samples.

**Figure 4:**
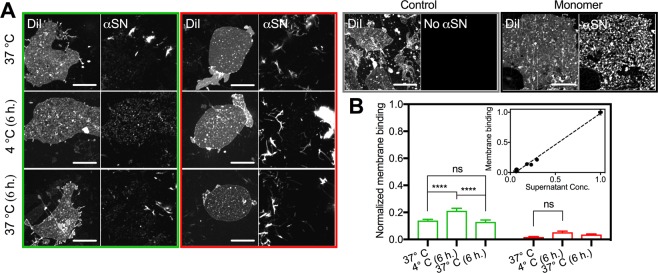
Structurally disordered species originating from early plateau fibrils interact with the plasma membranes. (**A**) CLSM images of membrane sheets incubated with early (green panel) or late plateau fibrils (red panel) immediately after extraction at 37 °C, after 6 h. incubation at 4 °C with or without subsequent incubation at 37 °C for 6 h. In addition we show sheets incubated with monomeric αSN prior to fibrillation (black panel) and a control without αSN (grey panel). Scale bars are 10 um (**B**) The quantified membrane binding (means ± s.e.m., n = 20) normalized to the binding before fibrillation (two-way ANOVA, Tukey Test; ****P < 0.0001, ns = non-significant). Inset shows the normalized membrane binding as function of the residual concentration of the supernatant.

It can be seen that the binding of the HMW and fibril αSN (bright structures in green and red panels of Fig. 4A) to the plasma membrane sheets is non-specific. This is concluded, as there is no preference towards the plasma membrane over the poly-ornithine coated glass surface. In fact, there is an apparent predominance of fibrillated αSN on the glass surface, which is explained by a higher glass/membrane ratio. This stands in contrast to the binding of monomeric αSN, which exclusively binds to punctate or wavy structures on the membrane sheets (Fig. 4A, black panel), and is not adsorbed onto the glass surface. Of interest, however, we note in the early plateau sample at 37 °C that a small portion of αSN still exhibits a binding pattern similar to that of monomeric αSN. This indicates a preservation of specificity towards the membrane sheets for these samples. Indeed, this correlates well with the observation that, at this point in the fibrillation pathway, there is still a significant amount of soluble and disordered protein present in the samples (Fig. 2a). Note, however, that this binding pattern has practically disappeared for the sample extracted from the late plateau. At this point only large aggregates and fibrils can be found scattered throughout the surfaces.

The most distinctive difference between the early and late plateau samples is seen after the incubation at 4 °C for 6 hours (Fig. 4A, left middle). Here, a markedly increase of the characteristic small and punctate structures on the membrane sheets can be seen for the sample from the early plateau, while that of the late plateau remains indiscernible from its counterpart at 37 °C. The quantified amount of specific αSN binding to the membrane sheets, normalized to the amount of monomeric binding prior to fibrillation, can be seen in Fig. 4B. The amount of αSN present on the membrane increases significantly from 14 ± 1% to 21 ± 2%, after the sample from the early plateau has been exposed to 4 °C, and equally decreases to 13 ± 1% after reheating to 37 °C. In contrast, the small increase in normalized membrane binding exhibited by the cold incubated sample from the late plateau is not statistically different from that of the samples at 37 °C. This is in agreement with the only minor disturbance in the SRCD spectra of the late plateau sample exposed to lower temperatures. Additionally, there is an interesting correlation between the heated, cooled and reheated samples from the experiment with residual concentration of the supernatants after centrifugation and those from the membrane binding experiments (Fig. 2b, Inset). We thus note with interest, that the species that are reversibly released from the early plateau samples correlate with the appearance of membrane active and specifically binding αSN species.

### Ongoing αSN fibril maturation has consequences

Our results, which are obtained using a distinctive and novel combination of methodological approaches, are highly interesting, when considering that different αSN strains, i.e. structurally different αSN fibril polymorphs, exhibiting differences in biological activity, also associate with different pathologies^17,23^. Here, we conclusively show, that there is a conversion between structurally different αSN fibril polymorphs with different thermodynamic behavior and distinct differences in biological activity. This conversion happens within only a few hours, under experimental conditions that are often referred to as ‘near-physiological’ (i.e. in PBS buffer and at 37 °C). Only one of the two forms releases soluble species upon mildly stressing conditions, and this directly correlates with the presence of specifically membrane active species. The current experiments do not reveal whether the interaction with the plasma membrane is cytotoxic, nor can we conclusively decide whether the observable specific, punctate interactions are formed by monomeric or (smaller) oligomeric αSN species, although our experiments do indicate that the released soluble fraction is a mixture of monomers and smaller oligomeric species. We can however firmly conclude that these species co-exist in a reversible and dynamic equilibrium with the fibril polymorphs present in the early phase of the ThT plateau, quite as it has been suggested for other amyloid material^48^. It is, however, also interesting to consider our results in the light of the recent cryo-EM structures of a truncated (residues 1–121)^49^ and full-length version of αSN, the latter elucidating the different structures of two different fibril polymorphs^50^. It is evident from these structures, that most of the amino acids, associated with the known human familial mutants, are situated directly on the interface between the two intertwining protofilaments^49,50^. Hence, these familial mutants would destabilize the fibril form. However, in the alternative polymorph, the protofilament interface has shifted, such that the position of these mutations no longer co-incides with the interface^50^. Evidently, given the different interfaces, these two fibril forms would also expectedly reveal different thermodynamic stability, and we speculate that similar re-arrangements of the fibril structure could underlie the differences between EP and LP fibrils, observed in this study. Since the familial mutants associate with early-onset Parkinsonism^51^, the structures could support our notion, that fibril instability associates with pathology. Indeed our observations are interesting in this context.

Our report has several additional consequences, which are relevant to consider. It is well-known that the plateau in ThT fluorescent assays by no means can not automatically be considered as a stable phase of fibrillation^44^. Our results elaborate significantly on this notion, showing that under the disputably most commonly applied experimental conditions for αSN fibrillation, the solution is depleted from soluble material accompanied by a structural transformation between different fibril polymorphs, notably while both the ThT-signal and macroscopic appearance of the fibrils remain stable. This emphasizes the importance of careful characterization of fibril samples prior to the subjection to further experiments (e.g. cellular assays), since there may be a marked difference both in the structural, thermodynamic and biological characteristics of fibrils, that are virtually indistinguishable both by ThT fluorescence and TEM. Indeed, this observation also impacts on the use of fluorescent assays for the development of advanced models of fibrillation kinetics^52^ and suggests that further, experimentally based insight is needed, before evaluating the significance of a given ThT signal at a given time point.

We show that early-stage αSN fibrils are reservoirs of membrane binding species, and that this fibril morphology can undergo a maturation process leading to very robust structures. During our previous work, we also highlighted that a structural maturation of wt αSN fibrils was noticeable by small-angle x-ray scattering and linear dichroism, while a familial αSN mutant (A30P) known to associate with early onset PD matured differently^21^. These notions have evident impact on the development of therapeutic strategies. While most current research focuses on the early structural changes from the native state to early amyloidogenic species, with the aim to control which polymorphs or strains are formed, our results suggest that such an approach may not be sufficient in all cases. Indeed the fate of already formed fibrils may impact on the biological consequences. Under the experimental conditions of our investigations, one of the fibril forms seems to be less of a biological culprit than the other, suggesting that therapeutic intervention stabilizing this more robust form could be a promising approach.

A remaining open question concerns the driving force of the observed structural maturation of the fibrils, happening at the plateau. What causes the conversion to a significantly increased content of β-sheet and corresponding structural integrity? An answer to this question may come from the well-known longitudinal clustering of fibrils^53^. While individual fibrils seemingly actively exchange soluble protein material with the surroundings, the interior of clustered fibrils may form a chemical micro-reactor, excluded from such active exchange with the solute, and these changes may enable the maturation process, and enhanced formation of the fibril β-sheets.

In conclusion, we have applied a novel experimental approach to investigate the structural and thermodynamic features of αSN fibrils, comparing fibrils from the early and late phases of the plateau that are visible in a ThT fluorescence assay. We conclusively show, that while there is a continuous incorporation of monomers into further fibril material during the plateau, there is an accompanying structural maturation of the fibrils taking place. While our data reveal that the early phase fibrils are a reservoir of soluble and membrane binding species, which reversibly release from the dynamic fibrils, the later phase fibrils exhibit an increased structural robustness, and does not release membrane active αSN. These observations have evident impact on the design of future investigations of αSN fibrillation, and may suggest novel therapeutic avenues.

## Methods

### Materials

All materials, unless otherwise specified, were purchased from Sigma-Aldrich.

### Expression and purification of αSN

Human αSN, cloned into the *Escherichia coli* vector construct pET11a, was expressed and purified as we have previously described^54^. After the last purification step, the protein was dialyzed three times against MQ-water, lyophilized and stored at −20 °C until usage.

### Sample preparation, fibrillation assay and thermal alterations

The lyophilized αSN was gently dissolved in ice-cold PBS buffer (20 mM KH_2_PO_4_, 150 mM NaCl, pH 7.4) before spin-filtration (0.22 μM, Merck) at 800 rpm at 5 °C. The concentration of the filtrate was determined by measuring the absorbance at 280 nm with a Nanodrop 2000 Spectrophotometer (ThermoFisher), and by using the sequence predicted extinction coefficient of 5960 M^−1^ cm^−1^ (ProtParam, ExPASy). A freshly prepared ThT stock solution in PBS was added to ensure a final concentration of 20 μM ThT and 50 μM αSN (0.7 mg/mL). The fibrils were produced in replicates of 180 μL in a 96-well optimal bottom plate (ThermoFisher), with each well containing a 3 mm glass bead for agitation. The plate was sealed with two layers of polyurethane amplification tape to minimize evaporation. Fibrillation was induced in a Fluostar Optima plate-reader (BMG labtech) at 37 °C with orbital shaking (300 rpm, 2 mm) in cycles of 280 s shaking and 80 s pause. The ThT fluorescence was recorded by excitation at 450 ± 5 nm and emission at 480 ± 5 nm. The plate reader was paused after approximately 72 hours, when the fluorescence signal was becoming stable and the early plateau fibrils were extracted. Similarly, after about 136 hours the assay was stopped and the late plateau fibrils were sampled. The samples were subjected to temperature alterations as means of testing their stability and the residual concentration of soluble protein was determined by centrifuging at 14,500 rpm for 1 hour at the corresponding temperature using a cooling centrifuge (Hermle Z 326 K). After carefully separating the supernatant from the pellet, the concentration was measured in replicates of 5 and normalized to the concentration of monomeric αSN, which had not been subjected to fibrillation.

The size distribution of the soluble protein species in the supernatants were investigated by means of native gel electrophoresis. Samples were prepared according to protocol and run on a NativePAGE 3–12% Bis-Tris gel for 90 minutes at 150 V. In addition, 100 μL of the samples were pipetted into a disposable microcuvette and measured on a Zetasizer Nano ZSP, Malvern Instruments. Each sample was equilibrated for 60 seconds before measuring with a scattering angle of 173° and averaging over 10 individual runs. Measurements were collected in replicates of three.

### Synchrotron Radiation Circular Dichroism (SRCD)

All SRCD data was collected on the AU-CD beam line at the ASTRID2 synchrotron in Aarhus, Denmark. The ellipticity was measured in the spectral range of 170–280 nm with a resolution of 1 nm. The measurements during the fibrillation assay were performed using a circular quartz cuvette and 3 scans were recorded and averaged. In the case of the thermal stability measurements of the early and late plateau fibrils, a periscopic chamber was used. This was done to ensure that high molecular weight species would remain within the beam path over the time course of the temperature ramp. A ramp cycled the temperatures 37–30–25–20–15–10–15–20–25–30–37 °C, with 10 scans recorded at every temperature (20 scans at the 10 °C midpoint). All spectra were buffer-subtracted, smoothed using the Savitzky-Golay algorithm^55^ and transformed to molar ellipticity using the equation $[\theta ]=\frac{\theta \cdot 100}{l\cdot N\cdot c}$, where θ is the reported ellipticity in mdeg, *l* is the path length of the cuvette in cm, *N* is the number of residues and *c* is concentration in mM.

### Transmission Electron Microscopy (TEM)

Samples were prepared undiluted and in a 1:50 dilution with PBS to facilitate an acceptable separation between individually fibrils. A routine protocol for staining was used: 3.5 μL of sample was placed on a Copper 400 mesh grid coated with Formvar/Carbon film (Electron Microscopy Sciences). 10 μL of MQ-water was added after 60 s and the grip was carefully aspirated using the edge of a filter paper. For negative staining, 10 μL of a 2% uranyl acetate solution was added and left for 30 s, before 2 × 10 μL MQ-water was carefully pipetted into the droplet. The grid was aspirated and left to dry for a couple of hours prior to imaging. The images were recorded at the Core Facility for Integrated Microscopy (University of Copenhagen, Denmark) using a CM100 TWIN Transmission Electron Microscope (Philips) equipped with a side-mounted Veleta Camera (Olympus) and processed in the iTEM software suite (Olympus).

### Cell culture and plating

HEK293 cells were cultivated in T-75 culture flasks using DMEM 1965 (25 mM HEPES, 44 mM NaHCO_3_) media supplemented with 10% FBS and 1% Penicillin-Streptomycin at 37 °C in 10% CO_2_. The cells were split and the media changed twice a week. Two days prior to the *in-vitro* membrane binding experiment, round coverslips were plasma cleansed, moved to 12-well culture plates and coated with a 0.1 g/L poly-L-ornithine solution before washing three times with sterile MQ-water. The cells were split and plated onto the coverslips at a density of 100,000 cells/mL. After two days in the incubator, the cells were ready for preparation of supported native membrane sheets by sonication.

### Preparation of native plasma membrane sheets

The native plasma membrane sheets were produced using an adapted version of the Wu and De Camilli protocol^47^. The cell media was removed and the cells were washed with ice-cold PBS. A brief sonication pulse (0.5 s, 20% output, 1.5 cm distance) using a Branson Sonifier 250 was applied in order to shear the apical and lateral domain of the membrane from the basal membrane. After washing with PBS, the coverslips (now containing the supported cell-membrane sheets) were transferred to an immuno-blotting box and incubated with 5 μM DiI for 10 minutes at 25 °C. The coverslips were rinsed and left with 200 μL PBS until the binding experiment to prevent dehydration.

### Binding of αSN to native plasma membrane sheets and Confocal Laser Scanning Microscopy (CLSM)

Before addition of the αSN samples, the native cell-membrane sheets were incubated with 200 μL blocking buffer (1 mg/mL bovine serum albumin in 5% goat serum) for 30 minutes to reduce any non-specific binding. Following another round of washing, 200 μL of sample was left to incubate on the coverslip for 1 hour. After three washing steps, the sample was fixed with 200 μL of a 4% paraformaldehyde (PFA) solution for 10 minutes. For detection of αSN on the sample, an anti-αSN monoclonal antibody (MJFR1, Abcam) with epitope mapped to residues 118–123 was used. The antibody (Ab) was covalently conjugated to Alexa Fluor 647 NHS Ester (Sigma Aldrich) and purified according to protocol. The concentration of the conjugate was determined to be 0.5 mg/mL with a labeling degree of 370%. The conjugate was aliqouted and stored at −20 °C. For immunolabeling, 200 μL of a 1:100 dilution in blocking buffer was left to incubate for 1 hour, before washing three times and fixing again with 200 μL of a 4% PFA for 10 minutes. The coverslips were rinsed and mounted on slides using ProLong Gold Antifade Mountant (ThermoFisher). Slides were left shielded from light at 6 °C until imaging. Imaging was carried out using a Zeiss LSM 510 inverted confocal laser-scanning microscope equipped with an oil-immersion Plan-Apochromat 63x/1.4 DIC objective. Dual-color images were obtained in a sequential setup using a 543 nm laser to excite the DiI (lipid membrane) and a 633 nm laser to excite Alexa Fluor 647 (anti-αSN Ab).

### Image visualization, processing and quantification

Image visualization, processing and analysis were carried out in ImageJ^56^. Regions of interest (ROIs) were created manually by drawing a selection over the full area of a supported cell-membrane sheet, found in the 565nm-channel (DiI, lipid membrane). The membrane binding of αSN was quantified as the mean intensity in the 647nm-channel (anti-αSN Ab) over this area. However, sections including large clusters of fibrils on the membrane were excluded from the ROI to prevent concealing the specific binding of smaller species. The values were background-subtracted (defined as the mean intensity of membrane sheets incubated without αSN but treated with anti-αSN Ab) and finally normalized to binding of monomeric αSN. The means were calculated from 20 replicates for each experimental condition.

## Supplementary information

Supplementary Information
