# Supplementary material for: Early Stage Alpha-Synuclein Amyloid Fibrils are Reservoirs of Membrane-Binding Species
Source: Sci Rep. 2019 Feb 11;9:1733. doi: 10.1038/s41598-018-38271-2 (PMC6370759; doi:10.1038/s41598-018-38271-2)
Supplement: Supplementary file 1 — Supplementary Information [file 41598_2018_38271_MOESM1_ESM.pdf]

Supplementary Information:

Early Stage Alpha-Synuclein Amyloid Fibrils are Reservoirs of Membrane-Binding Species

Thomas Skamris,[1,+] Carlotta Marasini,[1,+] Kenneth L. Madsen,[2] Vito Foderà,[,3] and Bente Vestergaard[*1]*

[1]Department of Drug Design and Pharmacology and [3]Department of Pharmacy, Faculty of Health and Medical Sciences, University of Copenhagen, Universitetsparken 2, 2100 Copenhagen, Denmark

[2]Molecular Neuropharmacology and Genetics Laboratory, Department of Neuroscience, Faculty of Health and Medical Sciences, The Panum Institute – Maersk Tower 7.5, University of Copenhagen, 2200 Copenhagen N, Denmark

[*]Corresponding authors: vito.fodera@sund.ku.dk, bente.vestergaard@sund.ku.dk

+These authors contributed equally

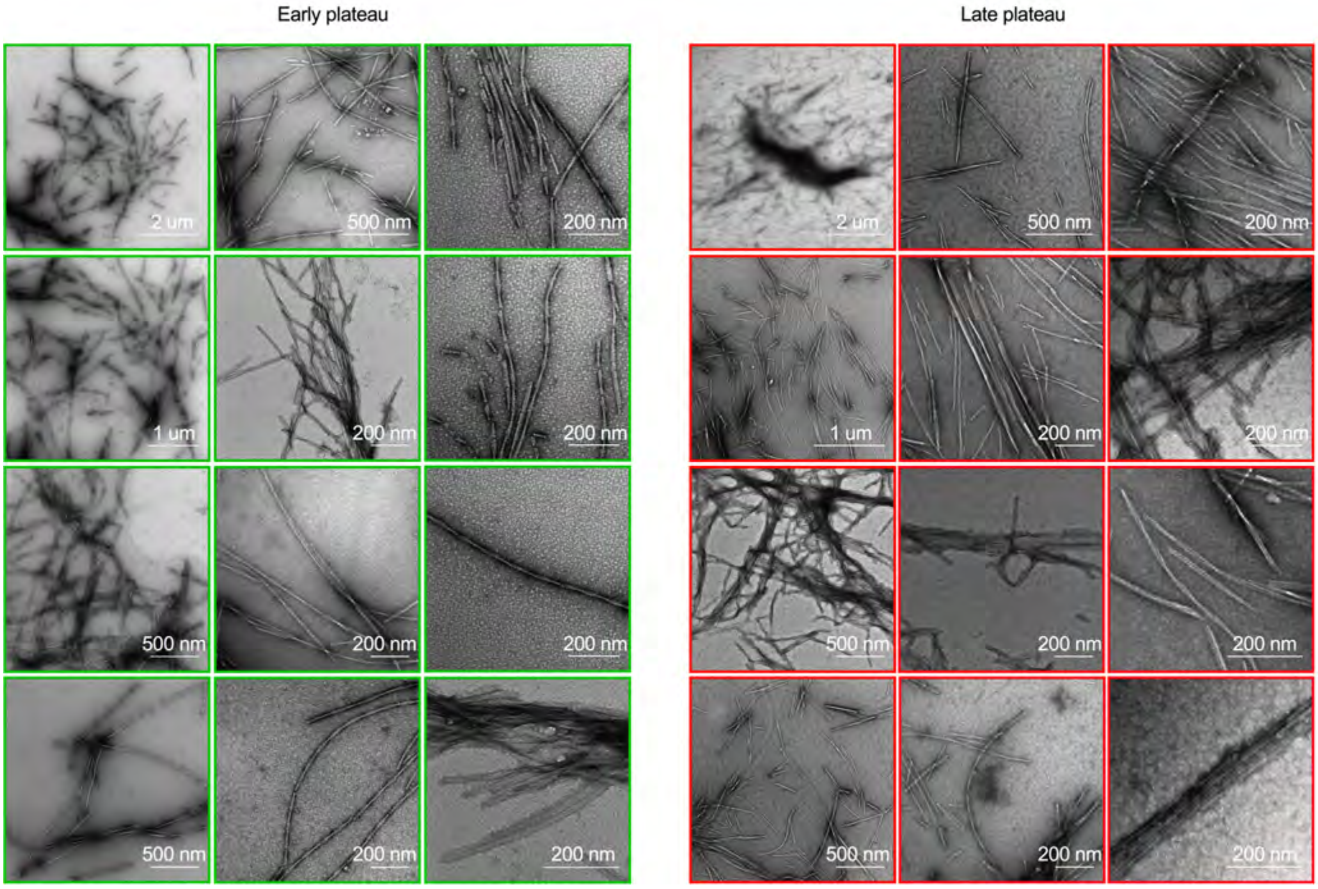

Figure S1. Fibril morphology and clustering of early and late plateau fibrils. TEM images of αSN amyloid fibrils from the early (green) and late (red) phases of plateau and taken at multiple magnifications. Lower magnifications show how both early and late plateau fibrils tend to assemble in clusters, while the high magnification images allow for morphological inspection of individual fibrils.

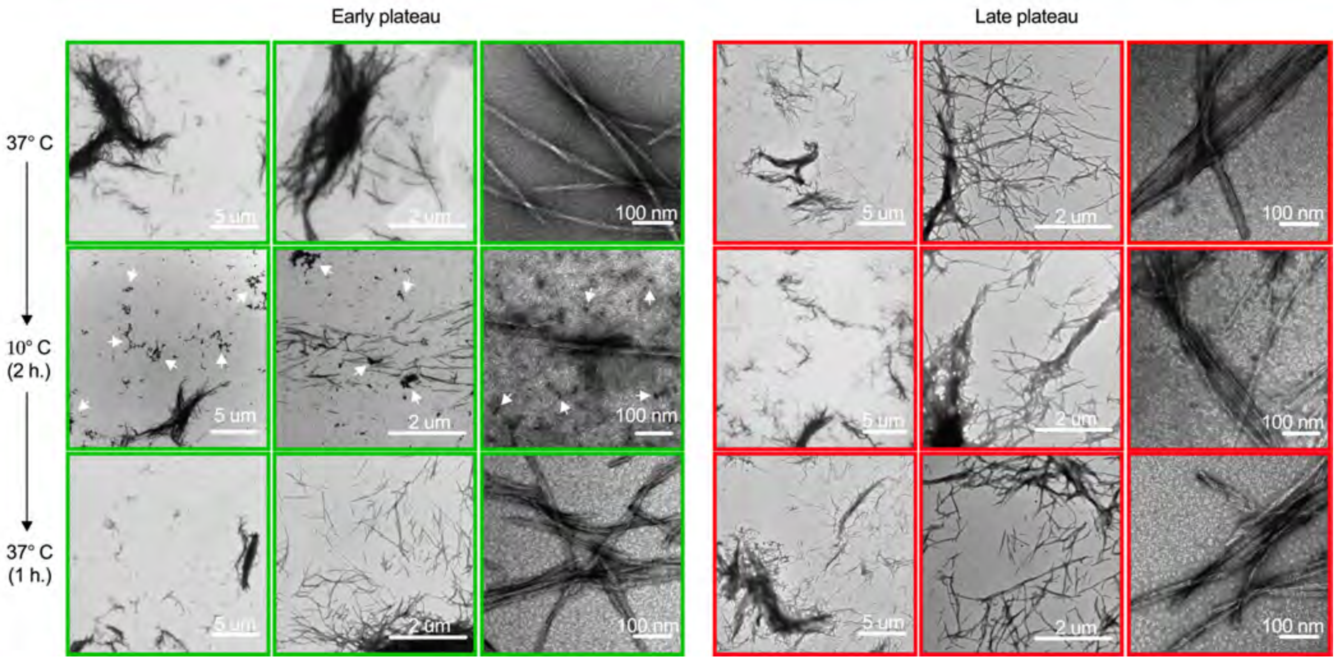

Figure S2. Reduced fibril clustering and amorphous aggregates observed for cooled early plateau fibrils. Collage of TEM images of αSN amyloid fibrils from the early (green) and late (red) plateau and subjected to alternating temperatures. The upper panel shows the fibril after extraction from 37 °C. The middle panel shows the samples after incubation at 10 °C for 2 hours and the lower panel is after reheating the samples at 37 °C for 1 hour. The arrowheads show the amorphous aggregates found after incubating the early plateau fibrils for 10 °C for 2 hours.

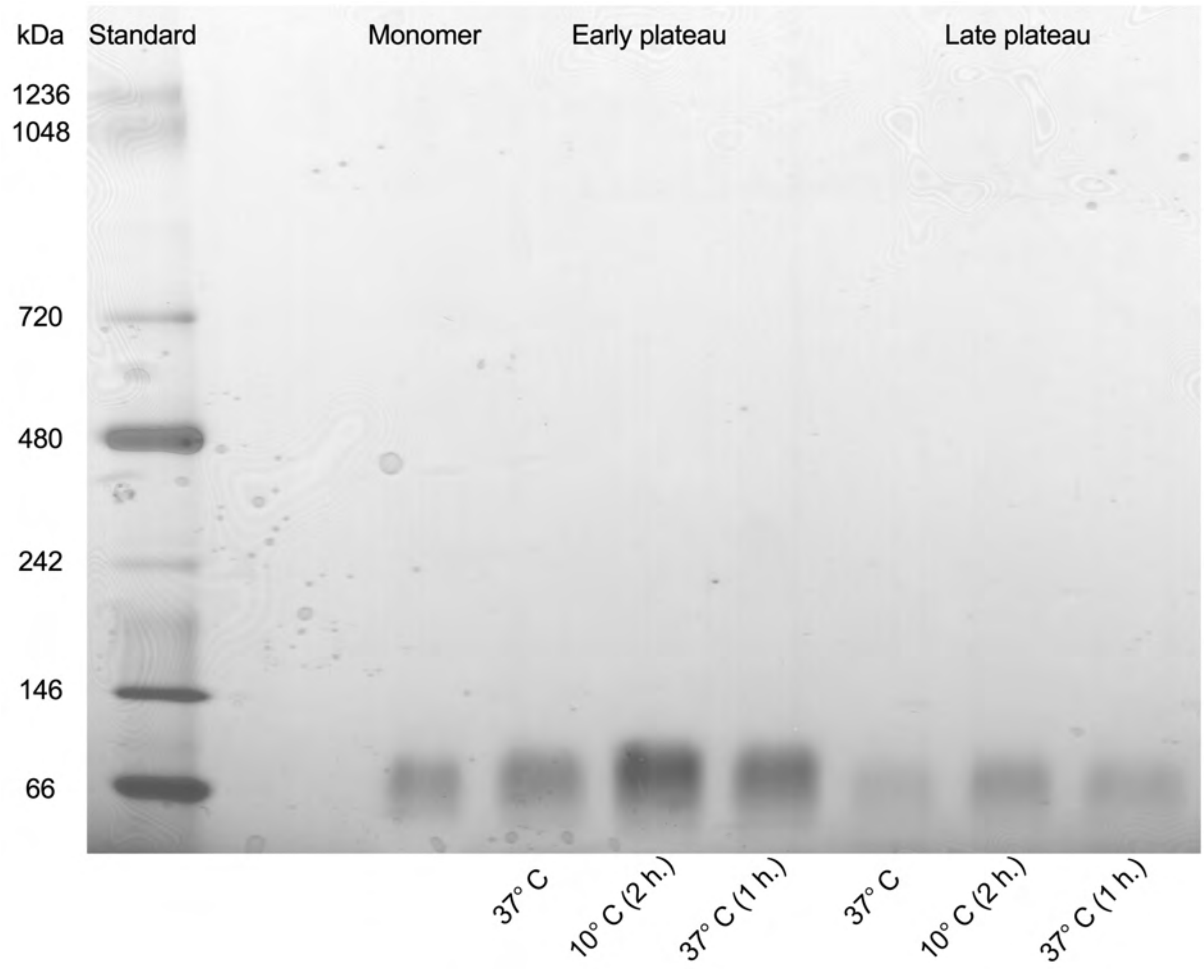

Figure S3. Cooling of early plateau fibrils releases protein species with a distinctive broadening in the migration profile. Native gel electrophoresis of monomeric αSN prior to fibrillation and of the supernatants from the early or late plateau subjected to different thermal alterations. The supernatants were produced by centrifugation for 1 hour at 14,500 rpm after direct extraction, and 2 hours incubation at 10 °C with or without subsequent incubation at 37 °C for 1 hour. A NativeMark standard ladder with reference proteins is added for comparison. The intrinsically disordered nature of monomeric αSN results in reduced migration distances compared to globular proteins of similar molecular weights. The gel is a single, uncropped gel.

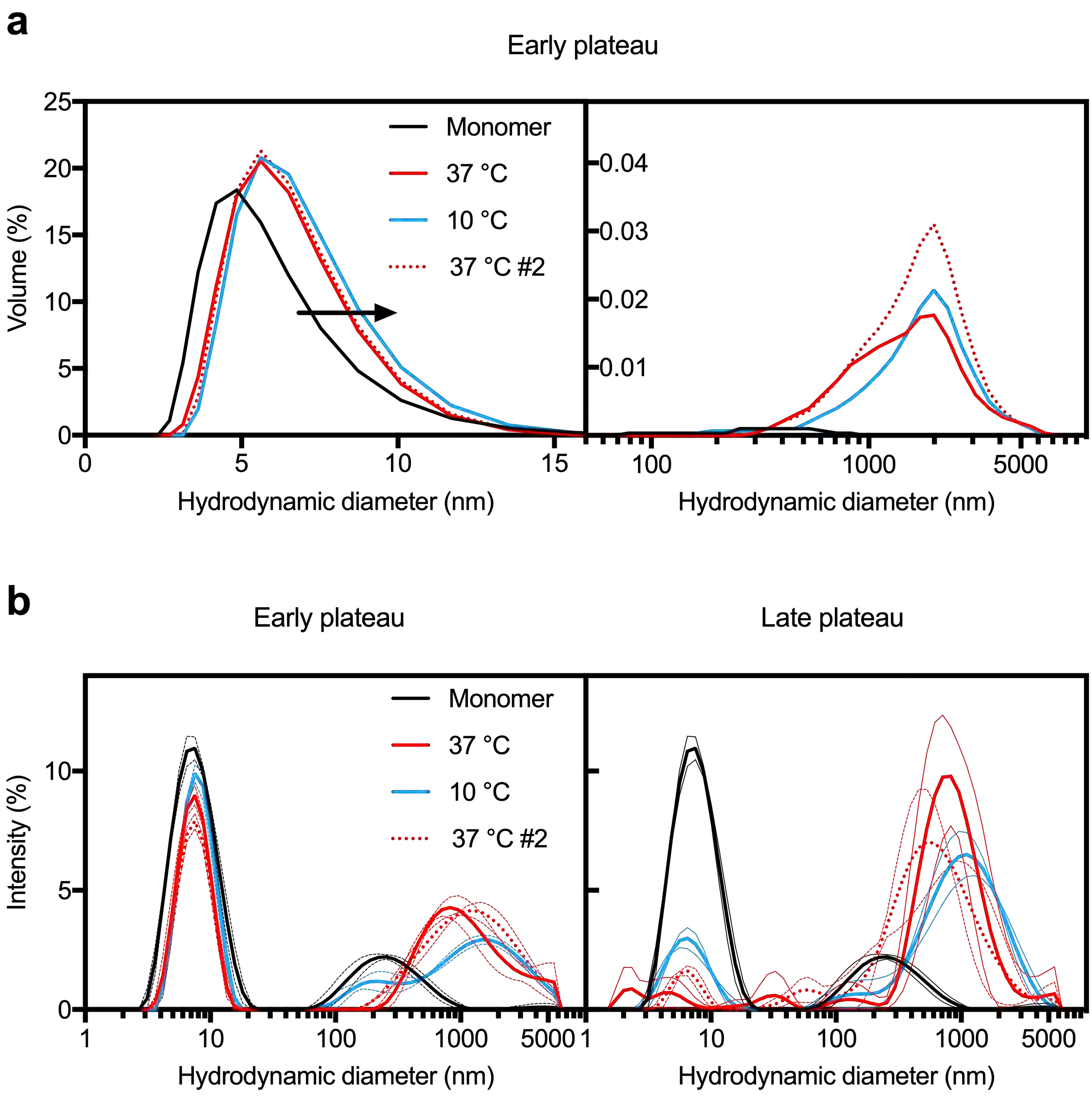

Figure S4. The early plateau samples contain soluble species with larger hydrodynamic diameters than the native monomer. (a) Volume-based particle size distribution (means, n=3) of monomeric αSN prior to fibrillation (black line) and of the supernatants from the early plateau after extraction (37 °C, red line), following 2 hours incubation at 10 °C (10 °C, blue line) and after reheating at 37 °C for 1 hour (37 °C #2, red stippled line). The left graph shows the shift towards larger particle sizes in the early plateau. The right graph reveals that only minute amounts of HMWS remain in the early plateau supernatants after centrifugation. (b) For comparison, the intensity-based particle size distributions (means ± s.d., n=3) of the supernatants for both the early (left graph) and late (right graph) plateau samples are shown, together with the data from monomeric samples. Colors are as in panel a). The amounts of

soluble protein remaining in the late plateau supernatants were too low for an accurate fit of the autocorrelation curve, hence rendering a proper particle size distribution analysis impossible.
